# Bases of Bacterial Sodium Channel Selectivity Among Organic Cations

**DOI:** 10.1038/s41598-019-51605-y

**Published:** 2019-10-24

**Authors:** Yibo Wang, Rocio K. Finol-Urdaneta, Van Anh Ngo, Robert J. French, Sergei Yu. Noskov

**Affiliations:** 1 Laboratory of Chemical Biology, Changchun Institute of Applied Chemistry, Chinese Academy of Sciences, Changchun, Jilin 130022 China; 2 Centre for Molecular Simulation and the Department of Biological Sciences, University of Calgary, Calgary, Canada; 3 Department of Physiology and Pharmacology, and the Hotchkiss Brain Institute, University of Calgary, Calgary, Canada; 4 Illawarra Health and Medical Research Institute, University of Wollongong, Wollongong, New South Wales Australia; 5 Center for Nonlinear Studies, Los Alamos National Lab, Los Alamos, NM 87544 USA

**Keywords:** Biophysics, Membrane biophysics, Permeation and transport

## Abstract

Hille’s (1971) seminal study of organic cation selectivity of eukaryotic voltage-gated sodium channels showed a sharp size cut-off for ion permeation, such that no ion possessing a methyl group was permeant. Using the prokaryotic channel, NaChBac, we found some similarity and two peculiar differences in the selectivity profiles for small polyatomic cations. First, we identified a diverse group of minimally permeant cations for wildtype NaChBac, ranging in sizes from ammonium to guanidinium and tetramethylammonium; and second, for both ammonium and hydrazinium, the charge-conserving selectivity filter mutation (E191D) yielded substantial increases in relative permeability (P_X_/P_Na_). The relative permeabilities varied inversely with relative K_d_ calculated from 1D Potential of Mean Force profiles (PMFs) for the single cations traversing the channel. Several of the cations bound more strongly than Na^+^, and hence appear to act as blockers, as well as charge carriers. Consistent with experimental observations, the E191D mutation had little impact on Na^+^ binding to the selectivity filter, but disrupted the binding of ammonium and hydrazinium, consequently facilitating ion permeation across the NaChBac-like filter. We concluded that for prokaryotic sodium channels, a fine balance among filter size, binding affinity, occupancy, and flexibility seems to contribute to observed functional differences.

## Introduction

**Figure 1:**
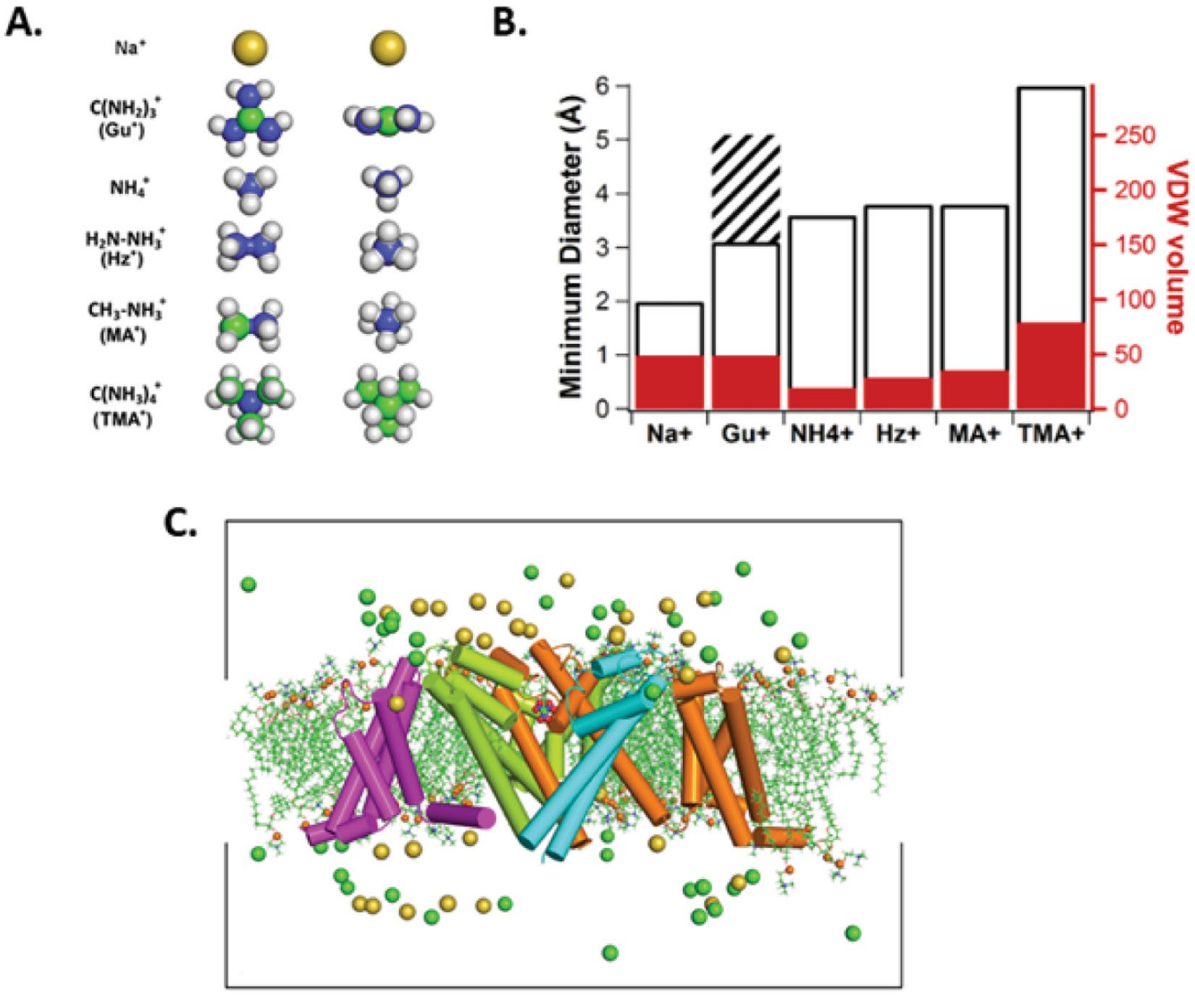
(**A**) CPK representation of organic cations: sodium (Na^+^), guanidinium (Gu^+^), ammonium (NH_4_^+^), hydrazinium (Hz^+^), methylammonium (MA^+^) and tetramethylammonium (TMA^+^). (**B**) Minimum diameters and van der Waals volumes for selected ions from the CPK models are presented. All bars should be read as starting from an ordinate value of zero (left axis, diameters; right axis, CPK volumes). The cross-hatched section for Gu^+^ indicates diameter across the planar section (5.1 Å) of the molecule, while the open bar reflects the “thickness” perpendicular to the plane (3.1 Å). (**C**) NavAb crystal structure embedded in a DMPC lipid bilayer. The nearest voltage-sensing domain and pore domain are removed for clarity. Chloride and sodium ions are shown as green and gold spheres, respectively. Lipid phosphorus atoms are represented by orange spheres, whereas the aliphatic sidechains are represented as sticks. The box provides the periodic boundary. The figure was adapted from the PhD thesis of one of the co-authors, Dr. Y. Wang^48^.

Small organic cations are useful probes to explore the molecular bases of the unique selectivity of different ion channel proteins. The permeability of polyatomic organic ions (Fig. 1A), as well as alkali cations, through voltage-gated sodium (Nav) channels has been the focus of numerous experimental studies (e.g.^1–3^) attempting to quantify ion selectivity. Hille’s (1971) study of the organic cation selectivity of Nav channels in myelinated nerves, showed a striking size-dependence for polyatomic ion permeation through the sodium selective permeation pathway. Subsequent experimental studies have often used impermeant polyatomic cations to block sodium channel currents in order to reveal the functional characteristics of potassium channel currents in excitable cells^4^. Hille proposed that, in frog Node of Ranvier, the sodium channel selectivity filter (SF) was lined by acidic residues that created a cation-selective pathway with a constriction as small as 3 × 5 Å^2^ in its cross section, that effectively “size excluded” the passage of larger polyatomic cations. It was later recognized that ion energetics based on physico-chemical properties were also determinants of selectivity^5–7^.

In the light of recent achievements in X-ray crystallography and Cryo-EM techniques, Hille’s hypothesis appears insufficient to account for the selectivity patterns associated with more recently observed structural diversity. Notable X-ray structures of bacterial Nav channels (NavBacs) implicate a wider and hydrated constriction for the cation sorting^8–11^. Moreover, NavBacs are formed as homotetramers and have a ring of EEEE in the selectivity filter, whereas eukaryotic Nav channel SF is lined by a DEKA ring in which each amino acid is contributed by one domain of a single polypeptide that folds into a pseudo-tetrameric structure^12^. The electrostatic environmental and steric constraints imposed by the NavBac selectivity filters are thought to be important factors determining relative permeabilities of specific alkali cations in Navs^10,13–16^. In the Cryo-EM structure of the eukaryotic (putative) Nav channel (NavPaS)^12^ from the American cockroach (*Periplaneta americana*), the DEKA selectivity filter is a few Å more constricted than its prokaryotic counterparts, nevertheless it is larger than Hille’s estimates for the frog node Nav SF. Despite of a relatively large selectivity filter, the sidechains of the acidic and lysine residues can stretch out 4–6 Å away from the backbone and appear to be sufficiently dynamic to reach out to either the opposite or proximal sidechains, effectively decreasing the cross-sectional area of the filter^17,18^, thus dynamically lowering ionic flux. In particular, movements of the glutamate sidechains appear to be able to create an efficient blockage by potassium ions^17,19,20^ directly supporting mechanisms proposed by French and collaborators in 1994^21^.

Furthermore, dynamic and ion-dependent conformational dynamics of acidic residues in the Nav SF (flipping) were proposed to play significant roles in catalyzing selective transport of Na^+^ ^22,23^. The combination of multi-microseconds long all-atom MD simulations with Markov-State analysis suggested that the liquid-like dynamical structure of the Nav SF can potentially be modulated by binding of different cations with conformational dynamics of acidic residues on the same time-scales as catalyzed ion movement^24^. This coupling between conformational transition in the selectivity filter and ion movement may also play a significant, yet not fully-understood, role in the well-established differences between inward- and outward-ion transport enabled by Navs^23^.

What remains unanswered is how these acidic sidechains help sodium channels to select or block different polyatomic ions. Answering this question would refine Hille’s hypothesis and provide a more accurate molecular basis of the polyatomic cation selectivity in prokaryotic sodium channels.

In order to address this issue, we compared the permeation of a panel of polyatomic organic cations with varied geometries and van der Waals volumes through NaChBac and its E/D pore mutant (Fig. 1A,B). This panel of organic cations is aimed at understanding the topology and electrostatic properties of the selectivity filters in both wildtype and mutant bacterial sodium channels^25^. We included tetramethylammonium (TMA^+^) and methylammonium (MA^+^) for comparison of the energetics of those with well-known, blocking polyatomic cations^26,27^. On one hand, TMA^+^ has been used widely as an external “inert or impermeant” extracellular substitute ion, which blocks either Nav or voltage-gated potassium (Kv) channels only from the cytoplasmic side. On the other hand, internally applied MA^+^ is probably the smallest organic cation that blocks both Kv and Nav channels. We used the crystal structure of NavAb and an E/D mutant^11^ to understand how the sidechains of the acidic residues mediate permeation and selectivity. Thus, we performed both experiments and simulations to test a model for the polyatomic-cation selectivity mechanism (Materials and Methods).

## Results and Discussion

### Electrophysiology

**Figure 2:**
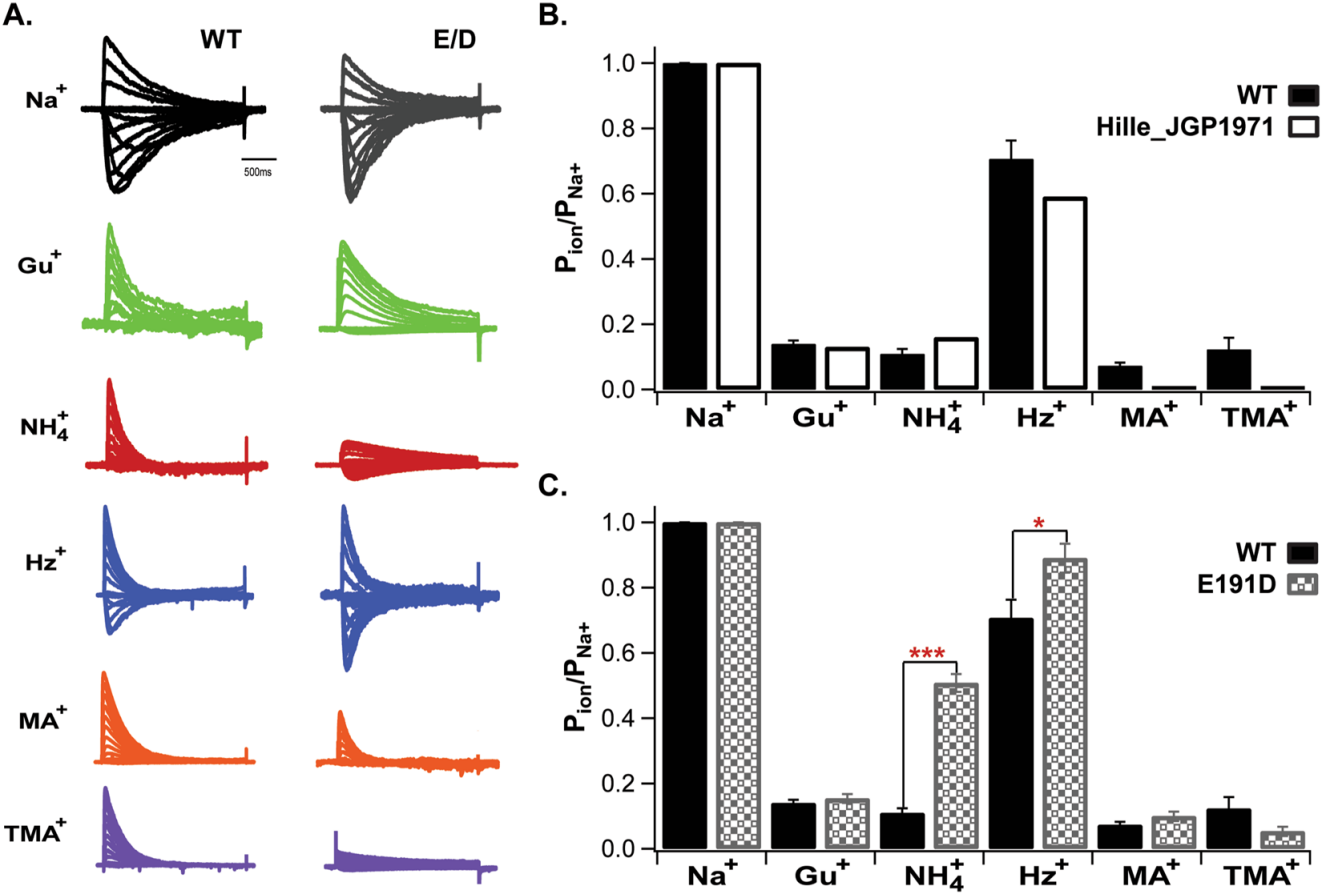
(**A**) Whole-cell NaChBac-mediated currents in the presence of different extracellular organic cations; (**B**) Relative permeabilities from I-V reversal potential shifts from NaChBac WT channels and from eukaryotic Nav channels as reported by Hille, 1971^1^; (**C**) Relative permeabilities (NaChBac WT and E191D) for selected polyatomic ions, calculated from reversal potential shifts following external monovalent ion substitution for sodium. For pairwise comparisons of WT vs E191D, P values are indicated as follows: *< 0.05; *** < 0.001. For the indicated comparisons.

To quantify the permeability of the polyatomic cations, we present patch-clamp recordings on the panel of polyatomic cations in Fig. 2, which summarizes the experimental data obtained from NaChBac whole-cell currents in control Na^+^, and in the presence of the various polyatomic species. Figure 2A shows that Gu^+^, NH_4_^+^, MA^+^, and TMA^+^ cannot translocate the selectivity filter of NaChBac as indicated by the negligible inward currents. For comparison, we also show Hille’s permeability ratios obtained from myelinated nerves (Fig. 2B). Figure 2B shows that the bacterial and eukaryotic channels are almost indistinguishable for Na^+^ and Gu^+^, but distinguishable for the other four cations. Interestingly, the permeabilities of both Gu^+^ and TMA^+^ are essentially the same in NaChBac, but they are markedly different in the eukaryotic channel.

Based on a structure of the cockroach Nav channel, NavPaS^12^, one possibility is that the apparent large flexible loops extracellular to the selectivity filter may contribute to the recognition and exclusion of TMA^+^ ^28,29^, while their absence in the prokaryotic Nav structures may preclude chemical differentiation between Gu^+^ and TMA^+^ (Fig. 1A). An alternative possibility is that the eukaryotic outer vestibule is sufficiently rigid, and of appropriate shape, to exclude TMA^+^, but allow Gu^+^ to pass, consistent with Hille’s analysis (see Fig. 2B).

**Figure 3:**
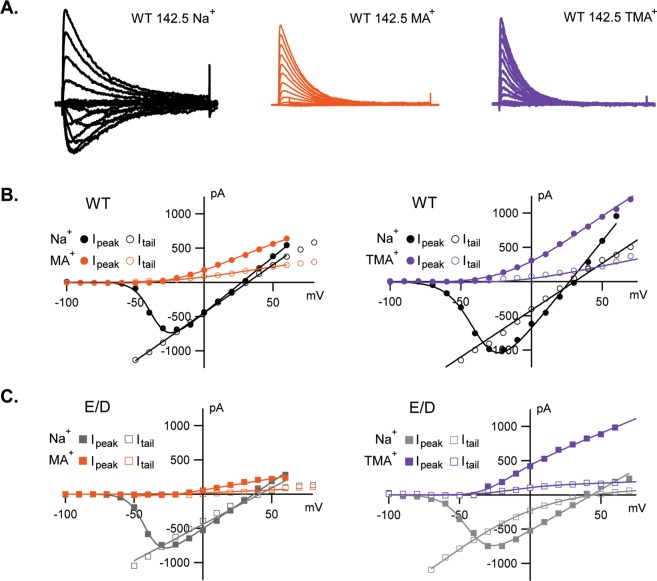
Inward currents carried by TMA^+^ and MA^+^ are almost undetectable. (**A**) Family of current traces resulting from a set of depolarizing pulses. Peak (filled symbols) and instantaneous (open symbols) I-V relations for NaChBac WT (**B**) and mutant E191D (**C**) in control Na^+^ and with TMA^+^ or MA^+^ in the external solution.

When E191 is replaced by D, there are not significant variations in permeability for Na^+^ or Gu^+^, but values for NH_4_^+^ and Hz^+^ change significantly (Fig. 2A,C). The permeability of NH_4_^+^ is increased 5-fold, while for the mutated channel E191D the permeability of Hz^+^ is equal to that of Na^+^. It is interesting that the permeability of TMA^+^ is reduced in the E191D mutant, closer to its impermeability in eukaryotic Navs. To better describe the differences conveyed by this mutation, Fig. 3 shows peak and tail currents in experiments where either TMA^+^ or MA^+^ was substituted for Na^+^ in the external solution, while the intracellular solution remained constant (see Methods). The outward currents (Fig. 3A), presumably carried by Na^+^ (Cs^+^ was found to be an unlikely charge carrier in our previous work^30^) from the pipet solution, show a slight reduction in TMA^+^ and MA^+^, suggesting a possible weak block of outward-moving ions when TMA^+^ and MA^+^ were present in the external solution.

Figure 3B shows the different blocking effects of TMA^+^ (purple) and MA^+^ (orange), relative to Na^+^ controls: WT (black) and E191D mutant (gray). MA^+^ reduces the sodium current amplitude more effectively than TMA^+^ in WT and E191D mutant NaChBac channels, in both peak and tail current measurements. This is clearly visible at zero voltage (0 mV). The data shows a stronger reduction in relative current with both MA^+^ and TMA^+^ for the E191D mutant than for the WT channel. Thus, the smaller MA^+^ ion appears to bind somewhat more strongly to the outer vestibule than does the larger species TMA^+^, and more strongly to E191D than to the WT channel.

### Permeation energetics

**Figure 4:**
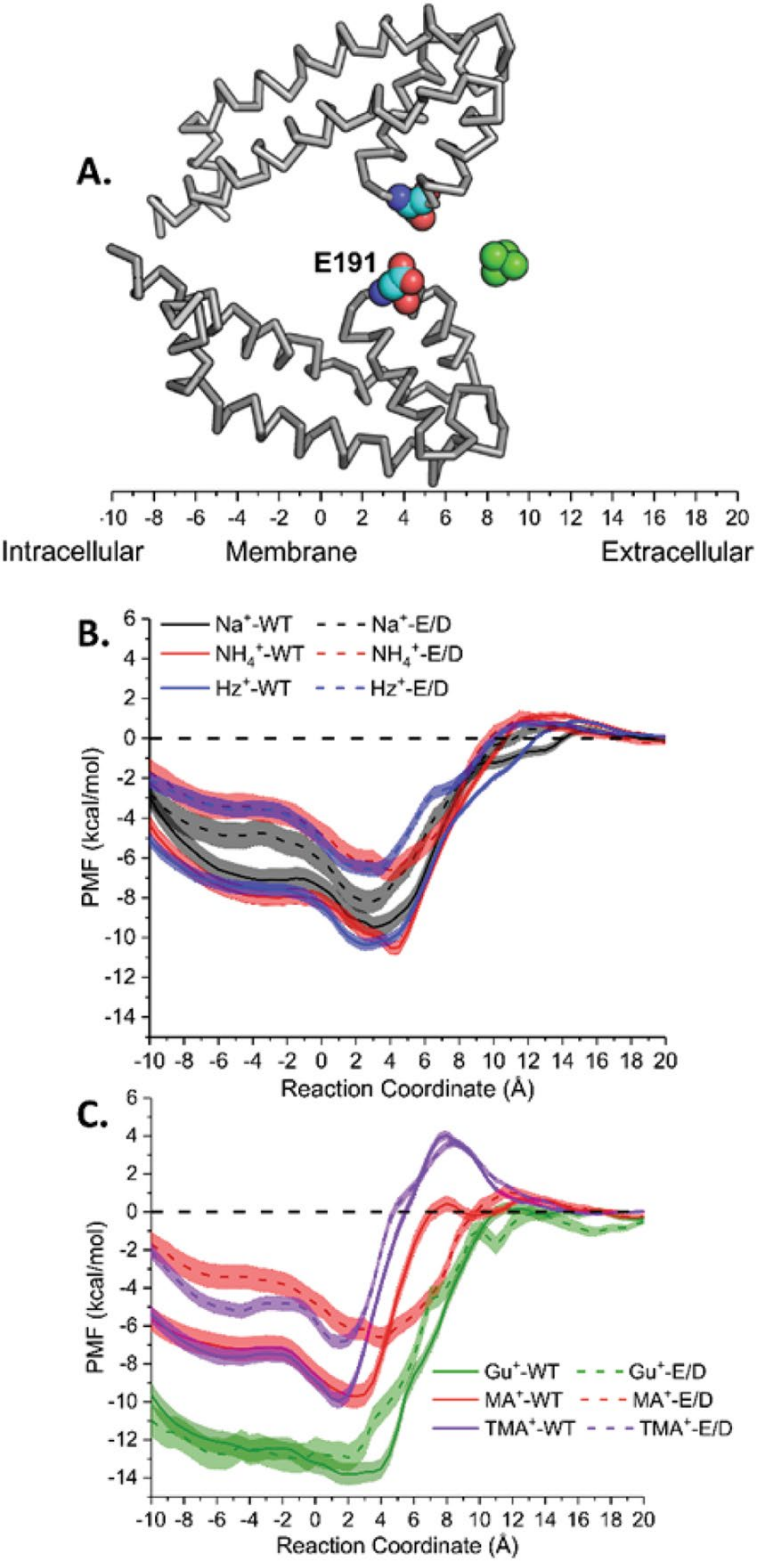
(**A**) Schematic of reaction coordinate for Potential of Mean Force (PMF) computations. The reaction coordinate is measured between the cations and the center of mass of the selectivity filter backbone atoms of residues 189, 190 and 191 (numbering corresponds to NaChBac sequence). Only two monomers are shown and the location of E191 is marked. An example of a permeant cation (NH_4_^+^) at one position along the reaction coordinate is illustrated; (**B**) 1D Potentials of Mean Force for permeant cations: Na^+^, Hz^+^ and NH_4_^+^; (**C**) 1D Potentials of Mean Force for Non-permeant cations: Gu^+^, MA^+^ and TMA^+^. 1D PMFs for cations in WT and E/D systems are shown in solid and dotted lines, respectively.

**Figure 5:**
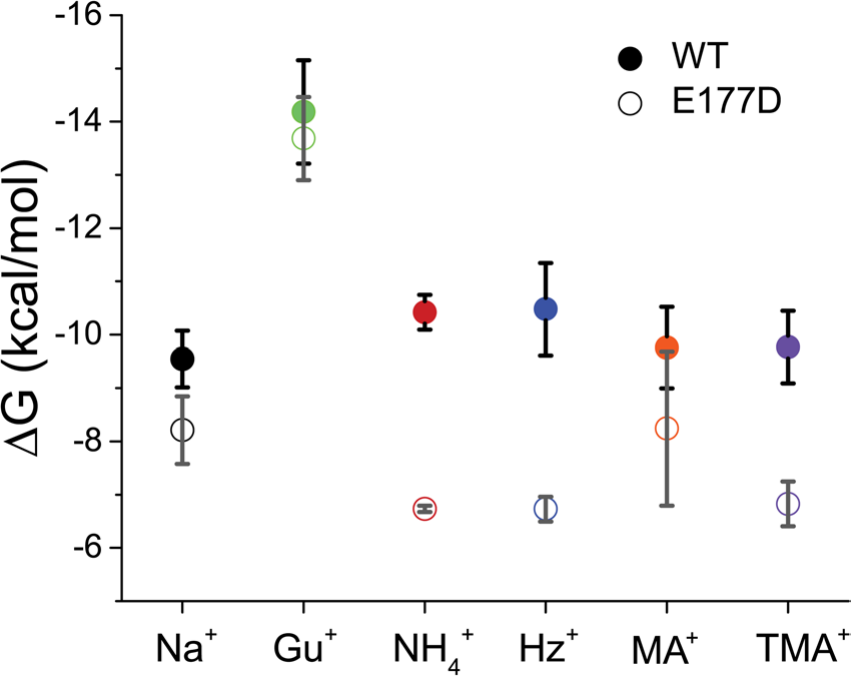
Cation binding free energies calculated from PMFs shown in Fig. 4B,C using Eq. (4) for the WT and E177D (E191D in NaChBac) systems are shown in solid and open symbols, respectively.

**Figure 6:**
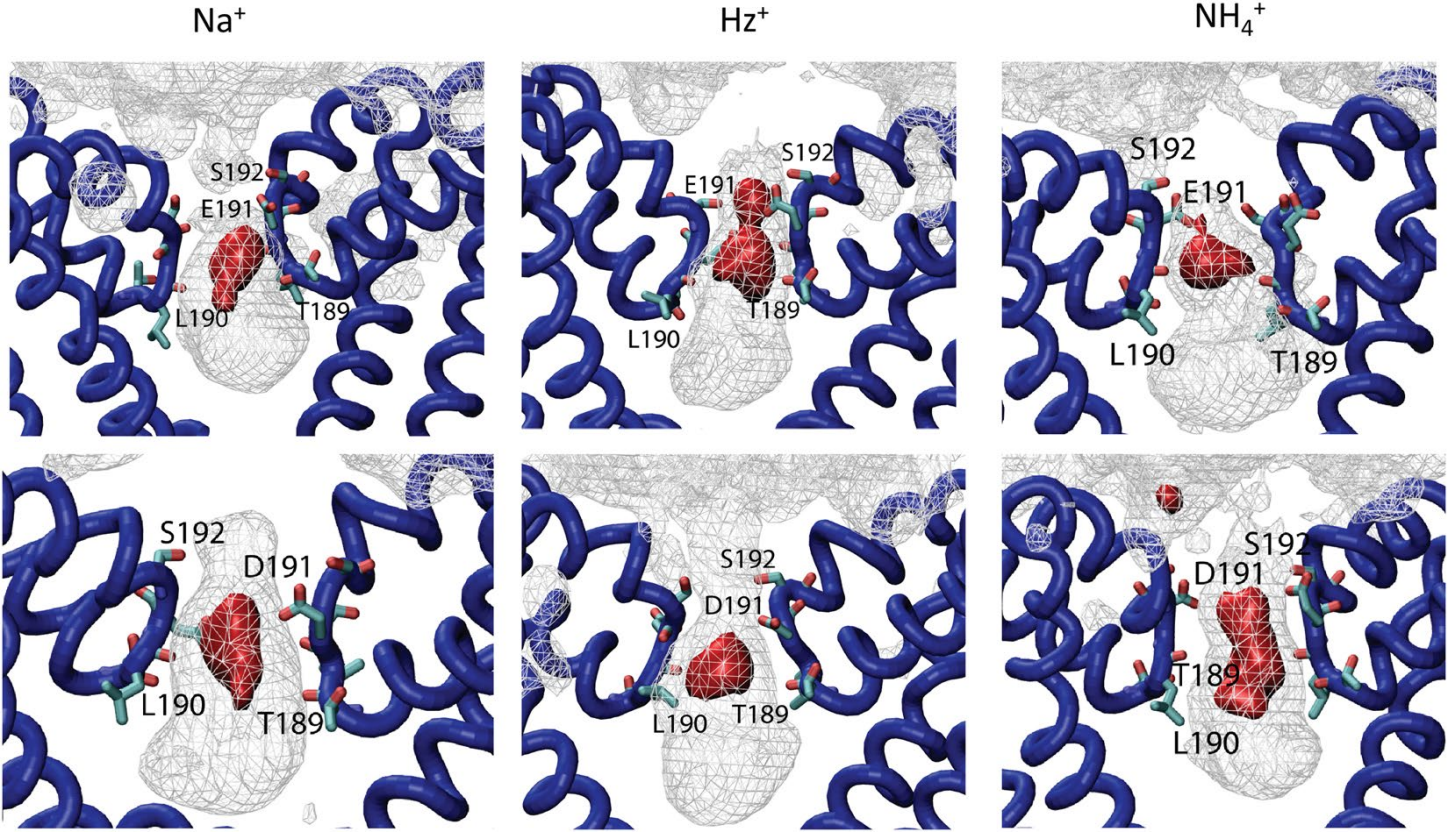
3-D particle densities (cation per Å^3^) for cations that are permeant across the Nav SF region for WT (E191 – upper panel) or mutant (D191 – bottom panel). For all polyatomic ions, the Centre-of-Mass position was used to plot binding volumes. The silver-colored density mesh illustrates region with density $({\rho }_{mesh})$ allowing weak binding with the affinity ~1 *k*_B_T relative to the bulk density (e.g. $\frac{{\rho }_{mesh}}{{\rho }_{bulk}}$ = 2.72), while the metal-red colored mesh corresponds to the regions with binding energy equal to, or exceeding, 3 *k*_B_T.

To explore possible factors underlying the observed permeation and block by polyatomic cations, we computed the PMFs of single cations with different contributions to their interactions with the SF of Navs (Fig. 4 – total PMF, Figs S1A and S2A – ion-protein, and Figs S1B and S2B – ion-water). From these data, we estimated equilibrium binding free-energies (Fig. 5). To aid the discussion of the similarities and differences in the location of the main cation binding sites, we calculated the 3D particle densities averaged from the 200-ns equilibrium simulations are graphically summarized in Fig. 6. In this section, we will demonstrate how the cations affect the conformational dynamics of the selectivity filters, which in turn determine conduction dynamics of the cations.

Figure 4B presents PMFs for Na^+^, Hz^+^, and NH_4_^+^ to exemplify permeant cation behavior for WT and the E/D mutant. Remarkably, NH_4_^+^ and Hz^+^ seem to bind more favorably than Na^+^, judged solely by the affinity (depth of the binding well) for the WT-NavAb filter. These differences are also pronounced in the density maps obtained from the equilibrium MD simulations (Fig. 6). For instance, when compared to Na^+^, the equilibrium density maps for Hz^+^ indicate almost identical preferences for binding poses *in-* and *below-* the plane of carboxylates, which coincides with a broader binding region present in 1D PMFs (Fig. 4). The analysis of equilibrium occupancies of NH_4_^+^ shows almost no *in-plane* binding mode for this cation. The Hz^+^ binding pocket organization near E191 is reminiscent of what is found for single K^+^ binding to the selectivity filter of NavAb^17,30^. The binding site for Hz^+^ was notably shifted compared to the one observed for Na^+^ and is reminiscent to the binding pocket mapped for K^+^ in previously reported 1D PMF calculations^19,30^.

The charge-conserving E/D mutations have been shown to change the geometry of the binding pocket for Na^+^ and K^+^ centered at position 191, and to impact dynamical stability of the filter by reducing conformational dynamics of E191^19,31^. Interestingly, we found that E191D replacement destabilized the region binding of Hz^+^ and NH_4_^+^ (Fig. 4B) and increased their relative permeabilities (Fig. 2). Equilibrium MD simulations also highlight differences in the ion accessibility to the selectivity filter region of WT and E/D mutants. For instance, there is almost no *in-plane* coordination observed for Hz^+^ binding to D191 SF. In stark contrast, NH_4_^+^ showed an additional binding to the *in-plane* configuration (Fig. 6) in equilibrium MD simulations, indicating multi-ionic occupancy of the selectivity filter. Again, this is in qualitative agreement with the binding region broadening shown in the E/D mutant 1D PMF.

It is important to stress that virtually all the studied ions show binding wells in calculated 1D PMF near or at the locations marked by E/D-191 (WT and E/D, respectively). This finding is in excellent agreement with the results of equilibrium simulations and previous studies of Na^+^ and K^+^ binding to Nav SF^17,19^. 1D PMF decomposition from ion-protein interactions (Figs S1A and S2A) shows marked differences in cation interactions near the main SF binding site between WT and D191 mutants. The reduced conformational flexibility in E191D appears to have little impact on conductance for Na^+^ (Fig. 2), but enhances relative permeabilities for K^+^, Hz^+^ and NH_4_^+^. Figure 4C shows similar 1D PMF analysis for weakly permeant or non-permeant cations, which suggests blocking actions under the influence of experimental voltage protocols.

**Figure 7:**
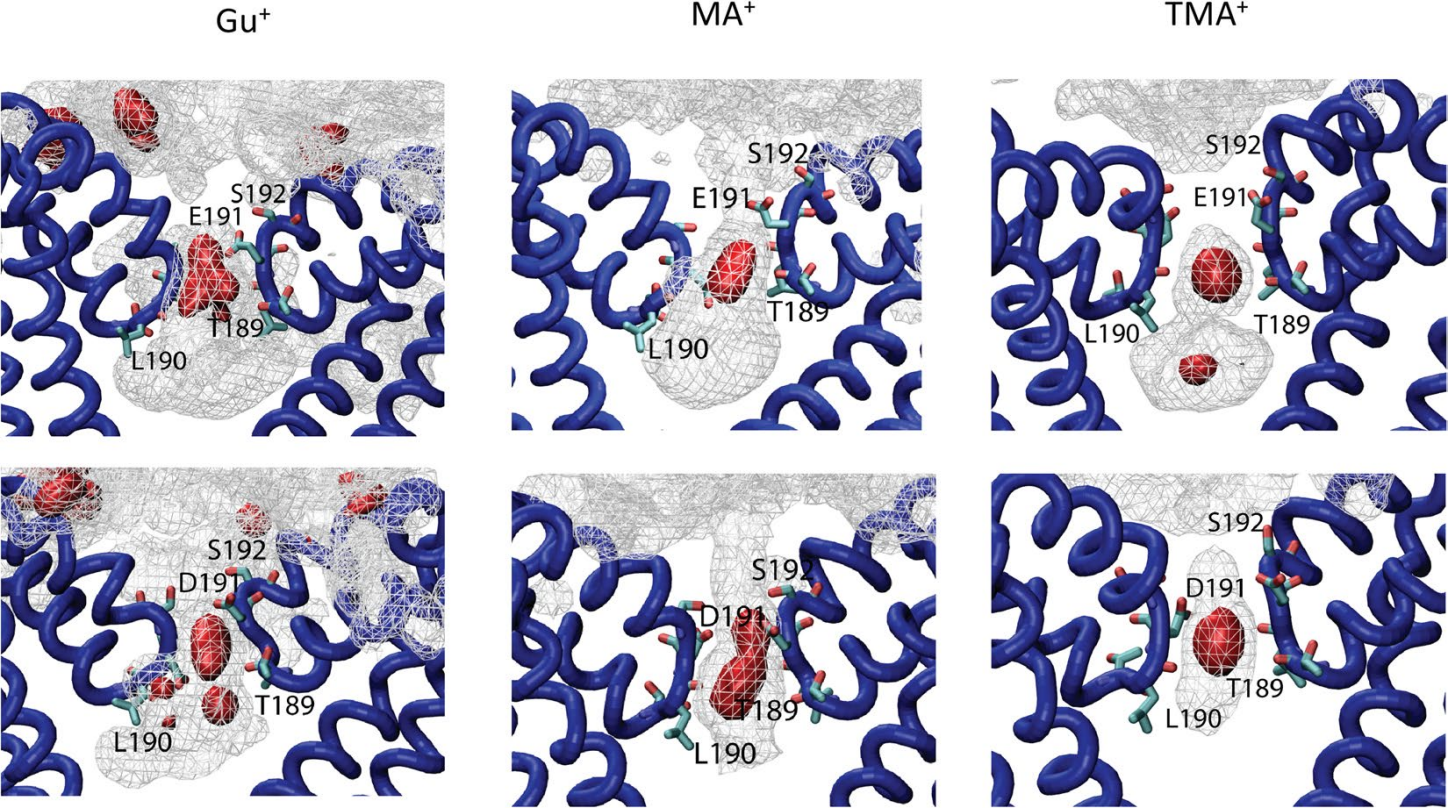
3-D particle densities (cation per Å^3^) for cations that do not permeate the Nav SF region for WT (E191 – upper panel) or mutant (D191 – bottom panel). For all polyatomic ions, the Centre-of-Mass position was used to plot binding volumes. The silver-colored density mesh illustrates region with density $({\rho }_{mesh})$ allowing weak binding energies of ~1 *k*_B_T relative to the bulk density (e.g. $\frac{{\rho }_{mesh}}{{\rho }_{bulk}}$ = 2.72), while the metal-red colored mesh corresponds to the regions with binding affinity equal or exceeding 3 *k*_B_T.

The 1D PMFs for Gu^+^ show the deepest binding wells among all studied cations (Fig. 5). This can be explained by the 3-fold symmetry of the cation and its cross-section allowing near-ideal interactions with the SF sequence in both WT and E/D channels. Binding free energies for Gu^+^ indicate its high intrinsic affinity (−14 kcal/mol) for the selectivity filter regions of both WT and the E/D mutant. In addition to a well-defined binding pocket located in the selectivity filter (S_CEN_), the Gu^+^ equilibrium densities (Fig. 7) display well-pronounced binding pockets in each of the protomers located on the extra-cellular surface of the channel marked by a ring of negatively charged glutamates (non-conserved D189 in NavAb replaced with an alanine residue in NachBac). The 1D-PMFs for MA^+^ and TMA^+^ in WT-NavAb present marked differences from all the other cations studied. These PMFs display high barriers combined with relatively-shallow binding wells at the selectivity filter region. It should be noted however, that their binding affinities (shown in Fig. 5) do not display significant differences from partially permeable cations like NH_4_^+^ or Na^+^. E/D replacement minimally impacts Na^+^, MA^+^ and Gu^+^ binding to the SF, but significantly destabilizes all other cations binding in the vicinity of E/D-191. The impaired binding of NH_4_^+^ and Hz^+^ results in the currents recorded for the mutant channel, while kinetic effects (barriers in 1D-PMFs) and steric factors appear to limit transport of MA^+^ and TMA^+^. These results indicate that NH_4_^+^ and Hz^+^ binding to the SF region are preferentially affected by the mutation.

The 1D-PMFs for permeant and non-permeant cations may not reveal the energetics of a full phase space of ion conduction manifested through possible multi-ion occupancies. To better understand general trends in multi-ion occupancies in Nav SF region, we performed series of 200-ns equilibrium simulations. The peak of ion densities for all studied systems is observed at the very broad binding area corresponding to the previously-described site S_CEN_. This binding pocket is marked by the side-chains of L190 and E191 (or D191), respectively. The S_CEN_ site has been characterized in great details in several previous studies featuring equilibrium and non-equilibrium MD simulations as well as various enhanced-sampling techniques^14,19,30,32–34^. It is important to note that favourable cation binding to this site was reported for different states (open or closed) of NavAb^33,35^, NavMs^34^ and NavRh channels^36^. In good agreement with previously reported MD simulations^22^, we observed rapid ion exchanges between extracellular and intra-cellular binding sites of the selectivity filter and the bulk milieu. This suggests that, for most of the studied cations, the chemical potential in the water-facing sites is very close to that of the bulk solution. In the case of TMA^+^, we observed rapid ion dissociation from the binding pocket formed by E191 and S192, within the first 25 ns. No re-binding events were recorded for TMA^+^ cations but for 2–3 water molecules that occupied the extra-cellular binding site for the duration of production runs. The equilibrium ion occupancies of Nav SF were 1.3 ± 0.5 and 1.1 ± 0.6 for Na^+^ and other permeant cations (NH_4_^+^, Hz^+^), respectively. The average equilibrium occupancies are in qualitative agreement with recently reported estimates from Callahan and Roux for Na^+^ permeation across a truncated NavAb pore^19^. These ion occupancies suggest that the 1D-PMFs likely include the most crucial energetic features underlying the differences in conduction among the cations studied.

Importantly, while the full permeation process across NavAb is known to rely on multi-ion occupancy^17,18,22,30,37^, using 1D-PMFs appears to provide an idealized “effective” cation binding profile, which captures essential features and provides qualitative correlation with certain experimental results. This may resemble the infinite dilution situation – a common reference state for thermodynamic models of membrane transport^38^. Thus, analysis of idealized models for single-ion binding to the Nav SF may still provide valuable insights into possible thermodynamic bases for cation selectivity in the situation where any additional ion would be highly unlikely to enter the pore. For example, 1D-PMFs provide an appealing mechanism for the non-permeant Gu^+^ making it an apparent high-affinity blocker of both WT and E/D selectivity filters. Also important is that the presence of a second ion changes the free energy landscape in the filter of NavAb^17^, but apparently to a lesser, though significant, extent in eukaryotic Navs^18,21^. Nonethreless, the singly occupied state appears to be functionally important for ion transport process in Nav channels. The MSM analysis performed for multi-microsecond MD simulations of Na^+^ permeation across the NavAb filter suggests an alternating cycle among states with single (~11%), double (~66%) or triple (~23%) ion occupancy. Transitions between different doubly-occupied states were seldom found, and most of the transitions observed required single-ion occupancy in the filter^22^. Furini and Domene reported even higher presence of a singly occupied state representing of ~40% of all functional states involved into Na^+^ permeation^24^.

Recently, the ion-specific changes in the conformational dynamics of E191 within the selectivity filter were proposed to be a key driver in cation-specific permeation across Navs with large and flexible SF regions. For example, non-equilibrium simulations of three K^+^ ions showed significant re-orientation of E191 side-chains allowing in-plane ion binding and stable coordination within the filter, effectively blocking the permeation. The upward and downward (or “dunked”) states of E191 are described by two side-chain dihedrals e.g. χ_1_ = 60–180° and χ_2_ = 0–90° and χ_1_ = 140–200° and χ_2_ = 290–330, respectively. We observe dunking dynamics of the acidic residue in the position 191 for all studied polyatomic ions, with the notable exception of Hz^+^ (see representative snapshots shown in Fig. S3). The analysis of the equilibrium MD simulations performed shows that ~11 and 6% of all data display inward-facing (dunked) conformation for WT and E/D SF, respectively. This finding is in good accord with previous studies performed at equilibrium and different biasing conditions^23,24^. The distribution of states, however, appears to show strong dependence on the polarity of membrane potential as well as the specific force-field used^19,23,24^.

## Conclusions

Organic cations have been used to probe the characteristics of ion channels since the 1970s. In our study, we combine experiments and simulations to investigate the bases of selectivity of NaChBac-like channels for five different organic cations. For permeant species, the permeability appears to depend on a fine balance between binding to the main site in the selectivity filter, and the heights of the entrance and exit barriers. For impermeable species like TMA^+^, they must surmount a large entrance barrier in order to enter the filter, while their binding profile near the selectivity filter generally resembles that of modestly permeable NH_4_^+^. Strong binding to the selectivity filter region, or a high exit barrier, may inhibit a cation from permeating and can cause it to block inward movement of other, less strongly bound, ions.

Our results suggest that the E191D mutation generally reduces the affinity of permeant organic cations to sites within the selectivity filter, resulting in noticeable changes of the relative permeabilities, as observed in experimental studies. The mutation reduces the depths of the free energy wells in 1D PMFs, most dramatically for cations NH_4_^+^ and Hz^+^ making them substantially more permeant in the mutant than in the WT channel. This implies an intricate relationship between the response of the channel (WT, or with a selectivity filter binding site minimally modified by E/D replacement) to the presence of the particular cation. It is tempting to conclude that the SF conserved in NavMS, NavAb, and several other bacterial Nav channels^11^, displays selective permeation mechanisms that rely on the coupling between cations and charged-residues, and the local conformational dynamics of the key side-chains. This dynamical “tuning” of the E191 region to the permeant cations was also observed in studies performed with 2D^20^ or 3D-PMFs^17,19^.

Yu *et al*.^39^ previously proposed a mechanistic framework in which a degree of “architectural” rigidity may give a rise to nuanced selective permeation sequences in potassium channels, which generally do not involve sidechains. Our study adds a valuable insight to that framework: a flexibility of acidic sidechains can regulate cationic/polycationic flows and selectivity in sodium channels. The importance of local ion-dependent deformation of selectivity-filter topology has been well established for several membrane proteins, including recent work on ryanodine receptors^40^, aspartate transporter Glt_Ph_^41^, clotting proteases^42^, Na/K pump^43^, and sodium-coupled secondary transporters^44^. Our work exploring various organic cations in the selectivity filter of NavAb/NaChBac provides additional evidence for this concept.

## Materials and Methods

### Electrophysiology

Electrophysiological recordings and data analysis were performed as described previous in Finol-Urdaneta *et al*.^30,45^. In brief, tsA 201 cells^46^ were transfected with DNA plasmids encoding WT or E191D mutant NaChBac channels using Polyfect (QIAGEN). The NaChBac-pTracer-CMV2 construct was kindly provided by D. Clapham (Howard Hughes Medical Institute, Children’s Hospital, and Harvard University, Boston, MA). Whole-cell patch-clamp recordings were made at room temperature with an Axopatch 200B amplifier (Molecular Devices). Patch pipettes were pulled from glass (Corning 8161, Potash-Rubium-Lead) to resistance of 1.5–2.5 MΩ. Recordings were made 18–24 hrs post transfection using control external solution contained (mM): 142.5 NaCl, 2 CaCl_2_, 2 MgCl_2_, 10 glucose, and 10 HEPES, pH 7.4. External ion replacement solutions were made by substituting 142.5 mM Na^+^ by Gu^+^, NH_4_^+^, Hz^+^, MA^+^or TMA^+^. Control intracellular (pipette) solution (“Na_in_”) contained (mM): 105 CsF, 35 NaCl, 10 EGTA, and 10 HEPES, pH 7.2. External solution changes were achieved by local superfusion of the replacement solution over the patched cell.

Data were analyzed using Clampfit (Molecular Devices) and Igor (WaveMetrics). Peak I-V curves were fitted according to:

1 $${I}_{(V)}=(V-{V}_{rev})\,\ast \,{G}_{max}/(1+{e}^{\frac{{V}_{half}-V}{{V}_{slope}}})$$

where *I* is the macroscopic current, *V* is the command potential, *V*_*rev*_ is the reversal potential, *G*_*max*_ is the maximal conductance, *V*_*half*_ is the half-activation potential, and *V*_*slope*_ is the slope factor (mV/e-fold). For weakly permeant external ions, the reversal potential is expected to occur in a negative voltage range, at which very little conductance is activated. Thus, in the presence of weakly permeant ions, a pre-pulse to −10 mV was applied to induce maximal activation, and *V*_*rev*_ was determined from the instantaneous I-V relation, measured from the initial point in the tail current decay after steps to a series of voltages encompassing *V*_*rev*_. In order to provide a more accurate and reproducible estimation of *V*_*rev*_, the relative permeability was calculated according to:

2 $${P}_{X}/{P}_{Na}={e}^{({E}_{X}-{E}_{Na})/(RT/F)}$$

where ${P}_{X}$ is permeability of ion X^+^; ${P}_{Na}$ is Na^+^ permeability; and $(RT/F)$ is 25.4 mV. E_X_ and E_Na_ are the reversal potentials for ion X^+^ and Na^+^, respectively. Net junction potentials were balanced to reduce the pipette current to zero before seal formation as reported in^30^. The apparent reversal potential shift was corrected for changes in net junction potential associated with external solution changes, using predictions from the JPCalc module^47^, Clampfit (Molecular Devices). All summary data are presented as mean ± SEM (n), where n is the number of independent determinations. Statistical significance was evaluated using unpaired Student’s t test; the criterion for significance was taken to be P < 0.05, unless otherwise stated.

### Molecular dynamics simulation

The complete details of theoretical methods were published as part of a PhD thesis submitted by Y. Wang^48^. Briefly, initial structure of NavAb was obtained from the X-ray crystallographic coordinates deposited to the Protein Data Base with PDB ID:3RVY^11^. The simulation system was constructed using CHARMM-GUI membrane builder protocol^49^. The NavAb channel was embedded into a pre-equilibrated DMPC lipid bilayer and solvated in a 104 × 104 × 80 Å^3^ box filled with TIP3P water (Fig. 1C). The whole assembly was bathed with 150 mM NaCl to ensure electroneutrality. All MD simulations and analyses were carried out by the program CHARMM 42b2^50^. The CHARMM-36 force-field was used with CMAP corrections applied to protein internal geometries^51–53^. The NavAb E177D (E/D, corresponding to E191D in NaChBac) mutant was built and equilibrated in CHARMM as described previously^30^. The constant area NPaT ensemble was used for all simulations with pressure set to 1 atm and temperature to 315 K. Long-range electrostatic interactions were treated by the Particle Mesh Ewald (PME) algorithm^54^. Non-bonded interactions were switched off at 10–12 Å. The systems were simulated with an integration time step of 1 fs in orthorhombic periodic boundaries. The system has been equilibrated for 25 ns prior to umbrella sampling simulations.

### Equilibrium simulations of the multi-ion SF occupancy

The equilibrium simulations exploring binding sites accessibility in various salt solutions were performed using the protocol described above. Briefly, each system (WT and E191D) was re-solvated in a 150 mM of MCl solution, where M was Na^+^, NH_4_^+^, Hz^+^, MA^+^ or TMA^+^. The initial system was based on the previously reported low-energy states of NavAb channel containing 2 monovalent cations bound to the selectivity filter with an additional cation bound to the intra-cellular cavity^22,55^. All the constructed systems were equilibrated for 50 ns and then subjected to the production run of 200 ns. The cation density maps were calculated then from 20,000 frames using cation center of mass positions and are illustrated in Figs 6 and 7.

### Force-field development

The quality of the force-fields is arguably the most important factor for the accuracy of our computations. Thus, we chose to develop new force-fields for all cations missing from the current release of the CHARMM-36 force-fields, including hydrazinium and methylammonium, using latest data on free energies of hydration and the protocol developed by Huang and Roux^56^. Other cations were modelled using CHARMM CGENFF force-field parameters^57^. Several of the organic cations parameterized specifically for this work are compatible with CHARMM-27/CHARMM36 CMAP force-fields^58^. The topologies and parameter files compatible with CHARMM or NAMD program packages are provided in the Supplementary Information. The cation parameters in the appended topology and parameters files are consistent with standard atom type definitions in the CGENFF. Briefly, partial charges were obtained by fitting with *ab initio* electrostatic potentials calculated with HF/6-31 G(d). The Lennard-Jones parameters for the organic cations were optimized against solute-water interaction energies scanning with HF/6-31(d) level of theory and fixed geometry for water probe corresponding to TIP3P model adopted by CGENFF development platforms. All the parameters have been corrected to reproduce experimentally-available hydration free energies^59,60^.

### Preparation of umbrella windows

To ensure convergence of our 1D PMF profiles and sampling of the bulk portion of the free energy profile we resorted to cylindrical boundaries preventing counterions interactions with permeant probe^48,61^. The restraining potential was also used to prevent other mobile ions (Na^+^ or Cl^−^) from entering the cylindrical boundaries. US sampling windows were spaced every 0.5 Å from +12.5 Å to −10 Å, resulting in 46 windows for each PMF computations. We prepared initial windows by placing a permeant ion across central axis of the channel. The lateral displacement of ion(s) was restrained to a cylinder of 10 Å radius and the central axis along the z-axis^61,62^. Next, 1D US simulations were performed along the z-axis with harmonic biasing potentials with a force constant of 10 kcal/(mol·Å^2^). The zero position along the z-axis is the center of mass of the C_α_ atoms of residues T175, L176 and E177 (or T189, L190 and E191 in NavAb channel). The reaction coordinate corresponds to a Z-component of the distance between the ion and the zero position (See Fig. 4A). The previously reported main binding site for Na^+^ ^22,24,30,37^ was located between 2 and 6 Å on the reaction coordinate chosen. The simulation time per window was set to 3.5 ns and only the last 3 ns were used to rebuild the initial free energy profile. Sampling of cation dynamics in the confinement of ion channel remains very challenging. However, several recent publications indicated dramatic increase in sampling convergence with H-REMD US method described below.

### Umbrella-sampling simulations with hamiltonian replica-exchange

The equilibrated systems were used for Hamiltonian Replica-Exchange simulations (H-REMD) in NAMD 2.11^63,64^. In essence, the H-REMD algorithm^65^ computes the total potential energies of all replicas and exchanges two adjacent replicas in the space of $\,{\xi }_{i}$ according to the Metropolis criterion every 1000 steps. Each H-REMD set of simulations has 64 windows and Metropolis-based exchange rates of 19–28% between successive windows. The number of H-REMD windows was increased to provide better exchange-rates/sampling near the barrier regions mapped from 1D US simulations. All PMFs from H-REMD in the main text were obtained with sampling time of 2.5–4 ns/window or 160 to 256 ns per 1D-PMF.

### Evaluation of equilibrium dissociation constants

The equilibrium dissociation constant *K*_*D*_ (single) from 1D PMF in presence of a cylindrical constraint can be expressed as follows^48,62,66^:

3 $${K}_{D}^{-1}(single)=\pi {R}^{2}{\int }_{{z}_{min}}^{{z}_{max}}{\rm{d}}z\,{{\rm{e}}}^{-w(z)/{k}_{B}T}$$

where *R* is the radius of the cylindrical restraint oriented normal to the z-axis with *z*_*min*_ = −10 Å, *z*_*max*_ = 21.5 Å and *N*_*A*_ is an Avogadro’s number. The *w(z)* was offset to zero for an ion in the bulk phase. Specific calculation details are provided in the PhD thesis by Y. Wang and previously published studies^48,55^.

The binding free energy is calculated according to

4 $$\Delta G=RT\,\mathrm{ln}\,\frac{{K}_{D}}{{C}^{0}}$$

where *C*^*0*^ is the standard concentration which is 1 M.

### Uncertainty analysis for computed PMFs

The 1D PMFs were rebuilt with the weighted histogram analysis method (WHAM)^67,68^, and the tolerance for WHAM was set to 10^−7^ kcal/mol. The statistical uncertainties were estimated according to Zhu and Hummer^69^ as described below. The collection of well-converged umbrella windows was used to seed additional US simulations with Hamiltonian Replica-Exchange. Relative entropy is calculated in order to verify that the error is not underestimated in the conventional block-average analysis (See Fig. S4). The relative entropy, *η*_*i*_, is used to check the agreement between the histograms observed from the simulation and the histograms predicted from the WHAM results:

5 $${\eta }_{i}=\int {p}_{i}^{obs}(x)ln\frac{{p}_{i}^{obs}(x)}{{p}_{i}^{WHAM}(x)}dx$$

where *p*_*i*_ is the probability in window *i*. Large values of relative entropy indicate that the samplings are inconsistent, and were used as a criterion for need, or lack thereof, of additional sampling. The uncertainty in the PMF was also estimated based upon the variance of the reaction coordinate *var*($\overline{{x}_{i}}$) in window *i* during umbrella sampling simulations^69^.

6 $$var[G(x)]={(k\Delta r)}^{2}\cdot \mathop{\sum }\limits_{i=1}^{\frac{x-{r}_{0}}{\Delta r}}var(\overline{{x}_{i}})$$

where *i* denotes the number of window, *Δr* the distance between the center of the neighboring windows and *k* the force constant (*k* = 10 kcal/mol*Å^2^). The variance of the reaction coordinate can be obtained from block averaging. The total sampling data *M* in each window simulation is divided into *N* (here *N* = 10) blocks of size *B*, so ${\rm{M}}=N\cdot B$

The average in each block is given as

7 $$\overline{{x}_{n}}=\frac{1}{B}{\sum }_{j=1}^{B}{x}_{j+(n-1)B}\,\,for\,n=1,\ldots ,N$$

And the corresponding variance *var*($\bar{x}$) is

8 $$var(\overline{{x}_{i}})=\frac{1}{n(n-1)}{\sum }_{n=1}^{N}{(\overline{{x}_{n}}-\overline{{x}_{i}})}^{2}$$

## Supplementary information

Supplementary Information

## Data Availability

The data sets generated and/or analyzed during the current study are available from the corresponding authors on request.
