# Author Correction: Bases of Bacterial Sodium Channel Selectivity Among Organic Cations

**DOI:** 10.1038/s41598-019-55306-4

**Published:** 2019-12-09

**Authors:** Yibo Wang, Rocio K. Finol-Urdaneta, Van Anh Ngo, Robert J. French, Sergei Yu. Noskov

**Affiliations:** 1 Laboratory of Chemical Biology, Changchun Institute of Applied Chemistry, Chinese Academy of Sciences, Changchun, Jilin, 130022 China; 2 Centre for Molecular Simulation and the Department of Biological Sciences, University of Calgary, Calgary, Canada; 3 Department of Physiology and Pharmacology, and the Hotchkiss Brain Institute, University of Calgary, Calgary, Canada; 4 Illawarra Health and Medical Research Institute, University of Wollongong, Wollongong, New South Wales Australia; 5 Center for Nonlinear Studies, Los Alamos National Lab, Los Alamos, NM 87544 USA

Correction to: *Scientific Reports* 10.1038/s41598-019-51605-y, published online 24 October 2019

In the original version of this Article, Yibo Wang and Rocio K. Finol-Urdaneta were omitted as equally contributing authors.

Furthermore, the Acknowledgments section in this Article was incomplete.

“This work was supported by grants from the Natural Sciences and Engineering Research Council (Canada) to S.Y.N. (NSERC RGPIN-315019) and the Alberta Innovates Technical Futures Strategic Chair in (Bio)Molecular Simulations. V.A.N. was financially supported by Postdoctoral Fellowships (AIHS and CIHR) during 2015–2018, and by LANL Director’s Fellowship during 2018–2021 (V.A.N.). The classification number for this publication issued by LANL is LA-UR-19-29893. The experiments and simulations reported here also featured in the doctoral thesis of Y.W. https://prism.ucalgary.ca/handle/11023/3077. All of the computations were performed on the West-Grid/ Compute Canada facilities, and the University of Calgary TNK and GlaDos clusters acquired with direct support by the Canada Foundation for Innovation and NSERC RTI awards.”

now reads:

“This work was supported by grants from the Natural Sciences and Engineering Research Council (Canada) to S.Y.N. (NSERC RGPIN-315019), and to R.J.F. (NSERC RGPIN-418658) and the Alberta Innovates Technical Futures Strategic Chair in (Bio)Molecular Simulations to S.Y.N. V.A.N. was financially supported by Postdoctoral Fellowships (AIHS and CIHR) during 2015–2018, and by LANL Director’s Fellowship during 2018–2021 (V.A.N.). The classification number for this publication issued by LANL is LA-UR-19-29893. The experiments and simulations reported here also featured in the doctoral thesis of Y.W. https://prism.ucalgary.ca/handle/11023/3077. All of the computations were performed on the West-Grid/ Compute Canada facilities, and the University of Calgary TNK and GlaDos clusters acquired with direct support by the Canada Foundation for Innovation and NSERC RTI awards. Preliminary experiments by Ahmed Al-Sabi suggested the interesting difference in permeation of ammonium and hydrazinium through wild-type NaChBac, and its mutant, E191D.”

This has now been corrected in the HTML and PDF versions of the Article.

