# Supplementary material for: Bases of Bacterial Sodium Channel Selectivity Among Organic Cations
Source: Sci Rep. 2019 Oct 24;9:15260. doi: 10.1038/s41598-019-51605-y (PMC6813354; doi:10.1038/s41598-019-51605-y)
Supplement: Supplementary file 1 — Supplementary Information [file 41598_2019_51605_MOESM1_ESM.pdf]

SUPPLEMENTARY MATERIALS

Bases of Bacterial Sodium Channel Selectivity among Organic Cations

Yibo Wang[1,2], Rocio K. Finol-Urdaneta[3,4], Van Anh Ngo[1,5], Sergei Yu. Noskov[1*] and Robert J. French [3*]

[1]Center for Molecular Simulation, Department of Biological Sciences; University of Calgary, Canada
[2]Laboratory of Chemical Biology, Changchun Institute of Applied Chemistry, Chinese Academy of Sciences, Changchun, Jilin, 130022, China
[3]Department of Physiology and Pharmacology, and the Hotchkiss Brain Institute; University of Calgary, Canada
[4]Illawarra Health and Medical Research Institute, University of Wollongong, Wollongong, New South Wales, Australia.
[5]Center for Nonlinear Studies, Los Alamos National Lab, NM 87544, USA.

Optimization of parameters for hydration free energy calculations

The values of the hydration free-energies for the cations in TIP3P water are listed in Table S1 in comparison to previously reported data. The free-energies of hydration were calculated via the Free-Energy Perturbation (FEP) method using the staged protocol of Deng and Roux [1] with separate calculations to assess electrostatic ($\Delta G^{a}_{\text{elec.}}$), dispersive Lennard-Jones (LJ) ($\Delta G^{a}_{\text{disp.}}$) and repulsive LJ ($\Delta G^{a}_{\text{repu.}}$) potentials.[1] The non-electrostatic LJ potential was separated into purely repulsive and attractive parts using Weeks, Chandler, and Andersen's (WCA) decomposition scheme The staging in FEP simulations, turning off and on the solute-water interactions, was achieved by using three coupling (or staging) parameters (s, ξ, and λ) implemented in the PERT module of the program CHARMM. The repulsive component of the hydration free-energy was obtained with the staging parameter s set to 0.0, 0.2, 0.3, 0.4, 0.5, 0.6, 0.7, 0.8, 0.9, and 1.0. The dispersive attraction was obtained with the coupling parameter ξ, which was varied from 0 to 1 by increments of 0.1. The electrostatic free-energy contribution was obtained using the coupling parameter λ, which was varied from 0 to 1 by increments of 0.05. FEP simulations corresponding to every value of the staging parameters were performed independently using protocols described previously.[2,3] All FEP runs were performed for both forward and backward perturbation. The periodic boundary condition correction (PBC correction) due to the surface potential of TIP3P water [4] in keeping with previously reported simulations by Lamoureux et al.[5,6] Each of the FEP windows was run for 500 ps of equilibration, followed by 2.5 ns of forward and backward simulation time for every amino acid mimetic in both water and vacuum. Weighted Histogram Analysis Method (WHAM) [7,8] was used to compute the components of the hydration free-energy shown in Table 1. All simulation systems used to calculate the hydration free-energy contained 216 TIP3P water molecules and one solute at the center of the simulation box. Positional constraints were applied

on the cation's center of mass. The electrostatic interactions were calculated using Particle-Mesh Ewald (PME) summation, with a coupling parameter of 0.34, 6th order spline for mesh interpolation, and cubic periodic boundaries. Non-bonded pair lists were maintained with a cutoff of 16 Å, and a real space cutoff of 14 Å was used for LJ interactions, which were truncated via an atom-based energy switch algorithm from 10 Å to 12 Å with LRCs applied.

Table S1: Absolute hydration free energies for cations.

Cation	ΔG_{exp} (kcal/mol)	ΔG_{calc} (kcal/mol)
Na+	-98.8	-100.1
NH_4^+	-80[a]	-81.5
MA^+	-73[a,b]; -74.9[c]	-77.6
Gu^+	−77.3[b]	-78.3
Hz^+	-83[c]	-81.5
TMA^+	-67 [a]	-69

a, Jorgensen, W. L., Ulmschneider, J. P., & Tirado-Rives, J. (2004). Free energies of hydration from a generalized Born model and an ALL-atom force field. *The Journal of Physical Chemistry.* b, *108*(41), 16264-16270; b. Kelly, C.P., Cramer, C.J. and Truhlar, D.G., 2005. SM6: A density functional theory continuum solvation model for calculating aqueous solvation free energies of neutrals, ions, and solute− water clusters. *Journal of chemical theory and computation*, *1*(6), pp.1133-1152.

c, Housecroft, C.E. and Jenkins, H.D.B., 2017. Absolute ion hydration enthalpies and the role of volume within hydration thermodynamics. *RSC Advances*, *7*(45), pp.27881-27894

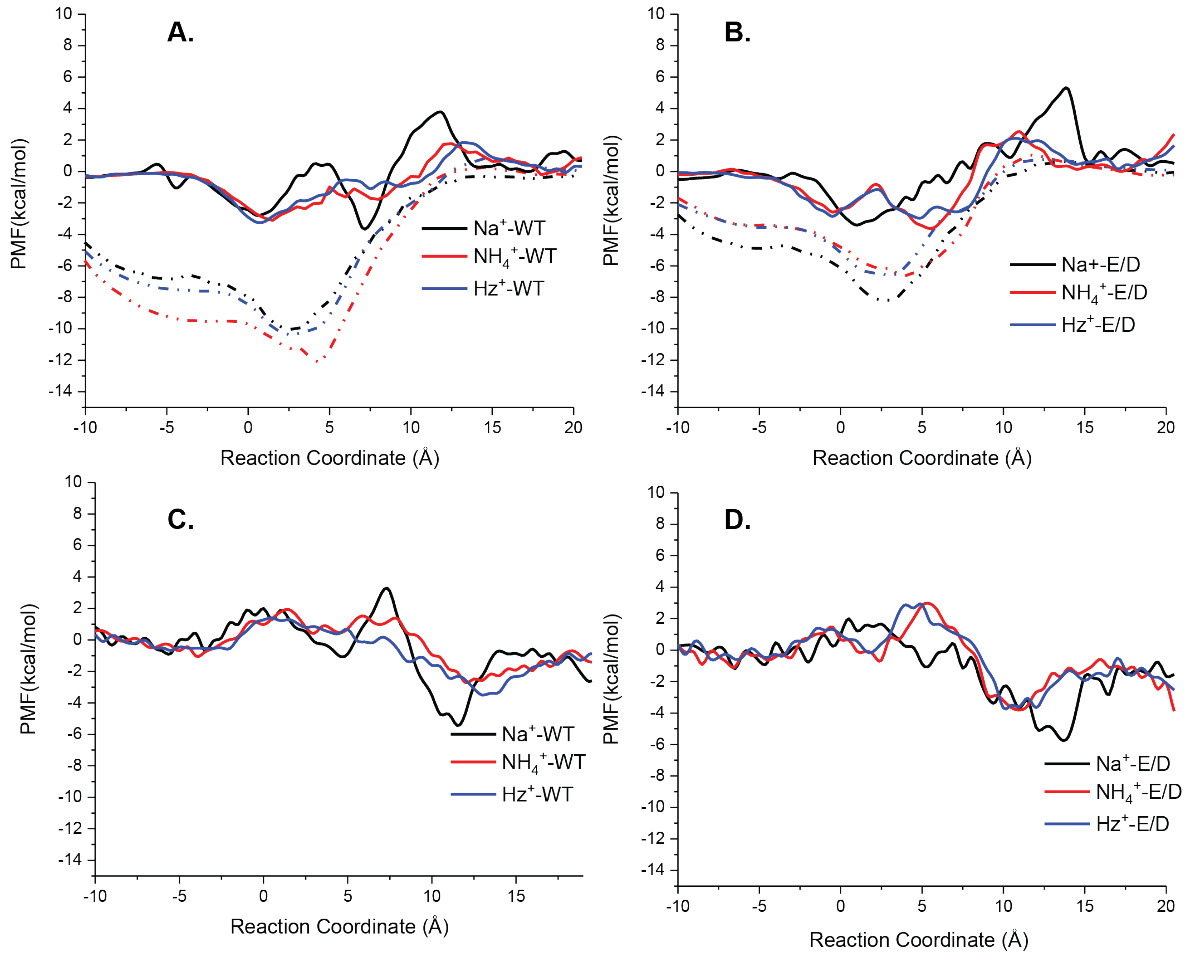

Figure S1. PMF Force Decomposition Analysis for Permeant Cations

Contributions from Ion-Protein (A and B) and Ion-Water interactions (C and D) to the total Potential of Mean-Force for permeant cations (Na^+, Hz^+, and NH_4^+) in WT and E/D systems. The corresponding total PMFs are shown as dash-dotted lines on the Panels A. and B. for comparisons. The decomposition analysis was performed with the method detailed in Allen et al.[9]

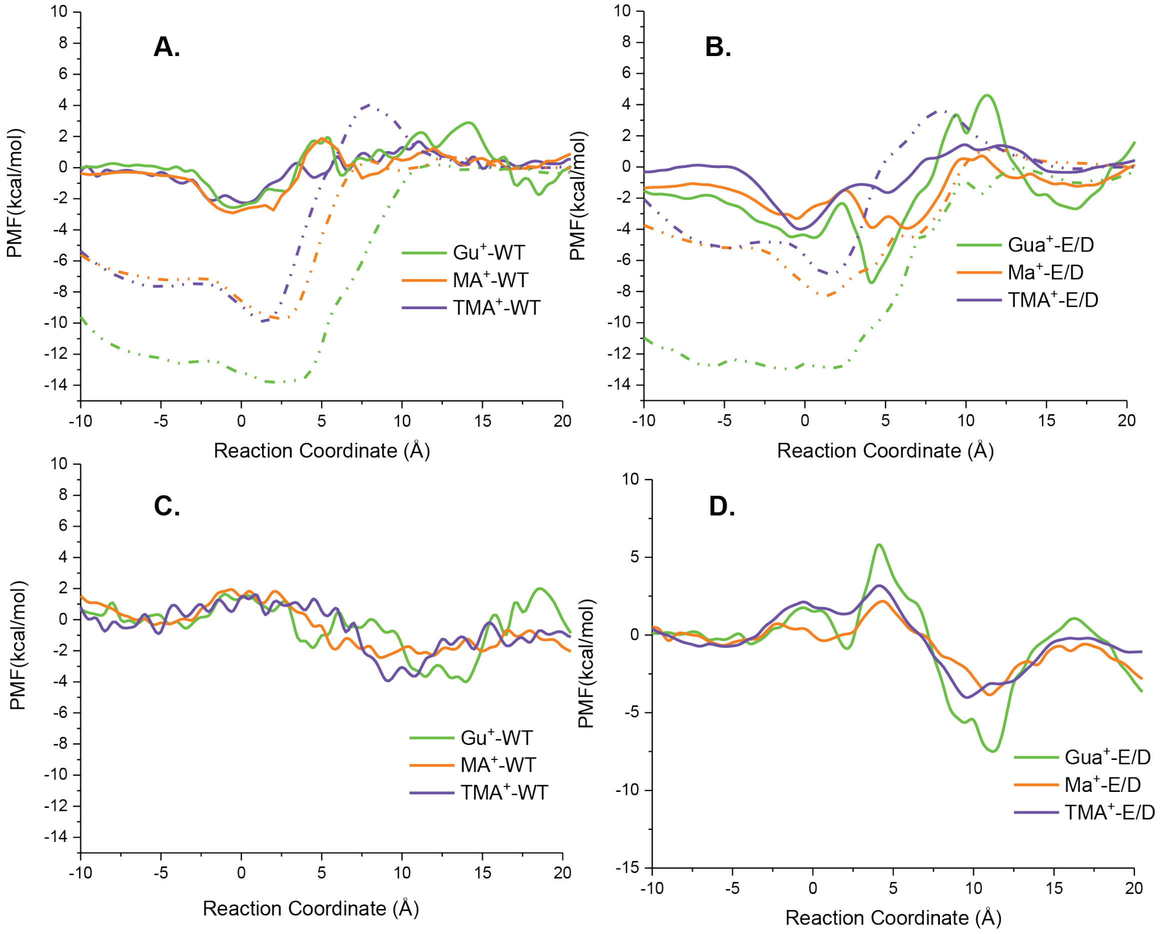

Figure S2. PMF Force Decomposition Analysis for Nonpermeant Cations

Contributions from Ion-Protein (A and B) and Ion-Water interactions (B and C) to the Potential of Mean-Force for nonpermeant cations (Gu^+, MA^+, TMA^+) in WT and E/D systems, respectively. The PMF components and total PMFs are shown in solid and dotted lines, respectively.

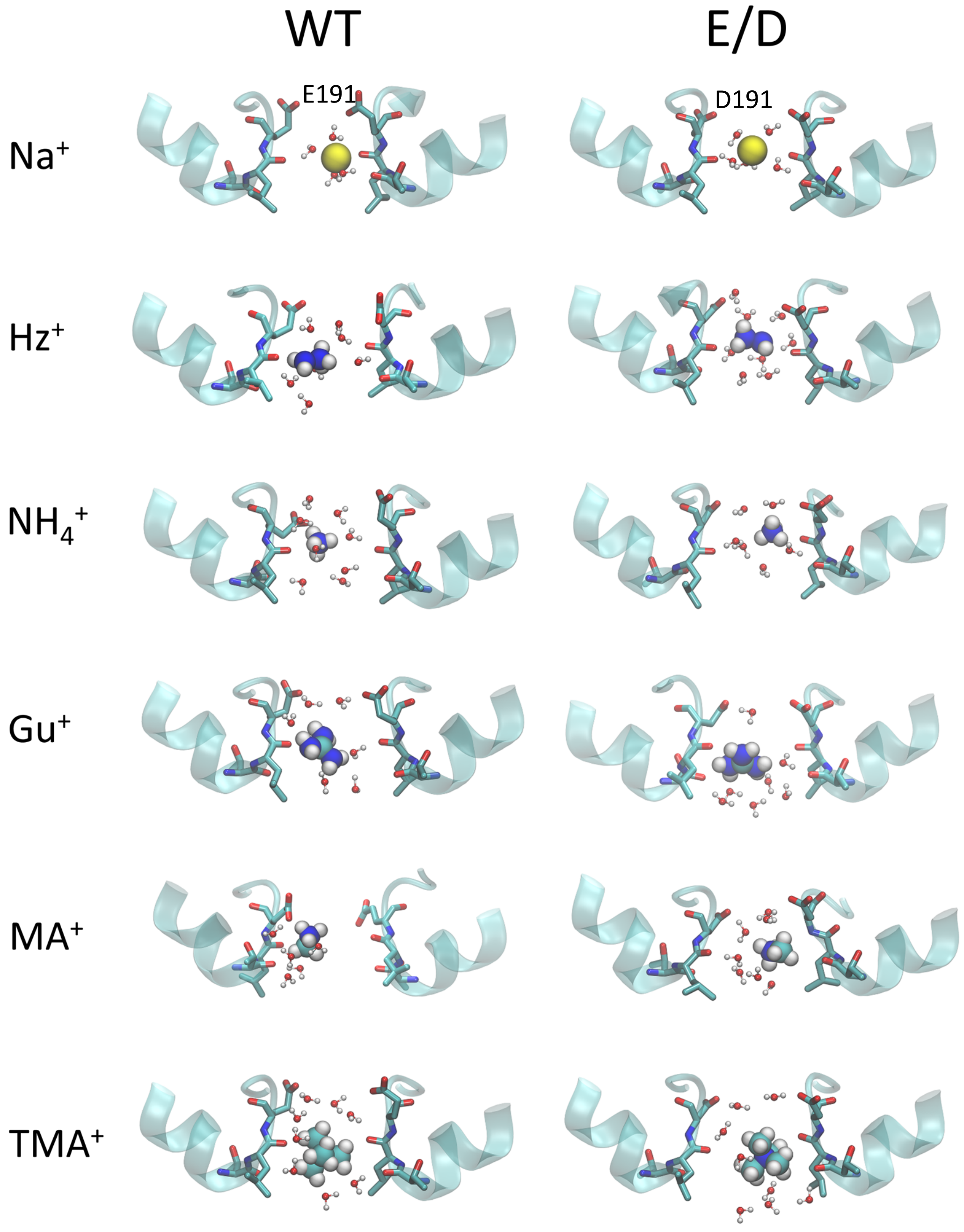

Figure S3.

The schematic illustration of the average structure of binding pocket corresponding to the minima in 1D PMF. Only two monomers are shown for clarity. The permeant ion is shown with a molecular sphere representation. The location of E191 or D191 is shown together with average number of water molecules within 3.5 Å within cation in the binding site.

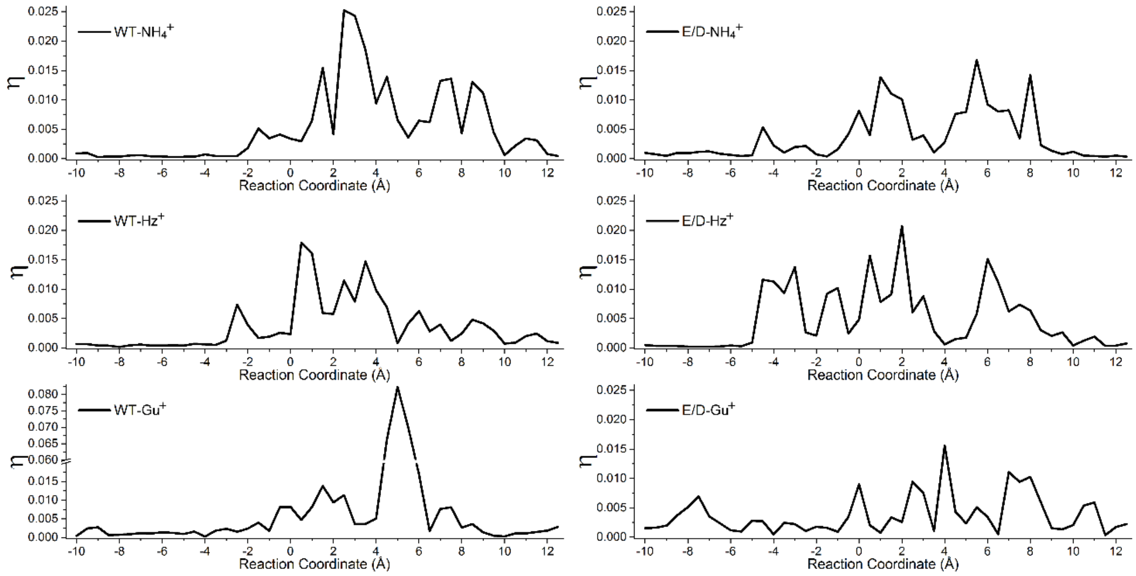

Figure S4

Relative entropy between the observed and WHAM histograms. The relative entropy values are all under 0.025 (except WT-Gu^+). We increased the simulation times for particular windows to keep the relative entropy at the same level for most of the organic cations. However, the value of relative entropy for Gu^+ in wild type posed particular difficulties. After 13 ns for window z = 5 and 5.5 Å, the relative entropy only decreased by 0.01. Converged windows were employed to build H-REMD simulations.

Supporting References

1 Deng, Y. Q. & Roux, B. Hydration of amino acid side chains: Nonpolar and electrostatic contributions calculated from staged molecular dynamics free energy simulations with explicit water molecules. *Journal of Physical Chemistry B* **108**, 16567-16576, doi:10.1021/jp048502c (2004).

2 Baker, C. M., Lopes, P. E. M., Zhu, X., Roux, B. & MacKerell, A. D. Accurate Calculation of Hydration Free Energies using Pair-Specific Lennard-Jones Parameters in the CHARMM Drude Polarizable Force Field. *Journal of Chemical Theory and Computation* **6**, 1181-1198 (2010).

3 Zhao, C. F., Caplan, D. A. & Noskov, S. Y. Evaluations of the Absolute and Relative Free Energies for Antidepressant Binding to the Amino Acid Membrane Transporter LeuT with Free Energy Simulations. *Journal of Chemical Theory and Computation* **6**, 1900-1914 (2010).

4 Lamoureux, G., Harder, E., Vorobyov, I. V., Roux, B. Æ. & MacKerell Jr, A. D. A polarizable model of water for molecular dynamics simulations of biomolecules. *Chemical Physics Letters* **418**, 245-249, doi:10.1016/j.cplett.2005.10.135 (2006).

5 Lamoureux, G. & Roux, B. Absolute hydration free energy scale for alkali and halide ions established from simulations with a polarizable force field. *Journal of Physical Chemistry B* **110**, 3308-3322 (2006).

6 Yu, H. B. *et al.* Simulating Monovalent and Divalent Ions in Aqueous Solution Using a Drude Polarizable Force Field. *J Chem Theory Comput* **6**, 774-786 (2010).

7 Roux, B. The Calculation of the Potential of Mean Force Using Computer-Simulations. *Computer Physics Communications* **91**, 275-282 (1995).

8 Kumar, S., Bouzida, D., Swendsen, R. H., Kollman, P. A. & Rosenberg, J. M. The Weighted Histogram Analysis Method for Free-Energy Calculations on Biomolecules .1. The Method. *Journal of Computational Chemistry* **13**, 1011-1021 (1992).

9 Allen, T. W., Andersen, O. S. & Roux, B. Molecular dynamics - potential of mean force calculations as a tool for understanding ion permeation and selectivity in narrow channels. *Biophys Chem* **124**, 251-267, doi:10.1016/j.bpc.2006.04.015 (2006).

Force field parameters for organic ions

Ammonium.str:

```
read rtf card
MASS   301 NAM   14.00700
MASS   302 HM     1.00800 AUTO ANGLES DIHE

RESI AMM         1.00
GROUP
ATOM NZ  NAM    -0.728
ATOM HZ1 HM      0.432
ATOM HZ2 HM      0.432
ATOM HZ3 HM      0.432
ATOM HZ4 HM      0.432
BOND NZ HZ1  NZ HZ2  NZ HZ3  NZ  HZ4
ANGLE HZ1 NZ HZ2    HZ1  NZ HZ3   HZ1 NZ HZ4   HZ2 NZ HZ3   HZ2 NZ HZ4   HZ3 NZ HZ4
IC HZ2  HZ4   *NZ   HZ1     1.0404 110.0200  120.2700 109.5000  1.0402
IC HZ3  HZ4   *NZ   HZ1     1.0404 110.0200 -120.1300 109.4000  1.0401
IC HZ1  HZ2   *NZ   HZ4     1.0402 109.4000  120.2700 110.0200  1.0402PATC FIRS NONE LAST
NONE

END

read param card
* MINI FORCE FIELD PARAMETER FILE.
*

BONDS
NAM   HM    403.9      1.010

ANGLES
HM   NAM  HM     44.000    109.50

DIHEDRALS
```

```
IMPROPERS

NONBONDED  E14FAC  1.000000
HM     0.000000  -0.100000    0.100000
NAM    0.000000  -0.170000    1.969900

END
```

Hydrazinium.str:

```
read rtf card
MASS   250 NZ2   14.00700
MASS   251 NZ3   14.00700
MASS   252 HZ     1.00800
AUTO ANGLES DIHE

RESI HYD          1.00
GROUP
ATOM N4   NZ3    -0.0784
ATOM H5   HZ      0.3182
ATOM H6   HZ      0.3182
ATOM H7   HZ      0.3182
ATOM N1   NZ2    -0.7142
ATOM H2   HZ      0.4190
ATOM H3   HZ      0.4190
BOND H5 N4 H6 N4 H7 N4
BOND N4 N1
BOND H2 N1 H3 N1
DONOR H5  N4
DONOR H6  N4
DONOR H7  N4
IMPR N4 H5 N1 H7
IMPR N1 H2 N4 H3
IC H6   N1   *N4  H5    0.0000  0.0000  120.0000  0.0000  0.0000
IC H7   N1   *N4  H6    0.0000  0.0000  120.0000  0.0000  0.0000
```

```
PATC FIRS NONE LAST NONE

END

read param card
* MINI FORCE FIELD PARAMETER FILE.
*

BONDS
NZ2   HZ    454.0       1.019
NZ3   HZ    421.0       1.028
NZ2   NZ3   324.0       1.452

ANGLES
HZ   NZ2  NZ3    65.00     108.08
HZ   NZ2  HZ     45.00     109.12
HZ   NZ3  NZ2    50.00     110.96
HZ   NZ3  HZ     45.00     107.84

DIHEDRALS
HZ   NZ2  NZ3  HZ      0.1565 3      0.0


IMPROPERS
NZ2  X    X    HZ        20.0000        0      0.0000
NZ2  X    X    NZ3       20.0000        0      0.0000
NZ3  X    X    HZ        20.0000        0      0.0000


NONBONDED  E14FAC  1.000000
NZ2    0.000000  -0.170000    1.890226
NZ3    0.000000  -0.170000    1.890226
HZ     0.000000  -0.015700    0.874959

END
```

Guanidinium.str:

```
read rtf card
MASS   267 HGP2     1.00800  ! polar H, +ve charge
MASS   291 CG2N1   12.01100  ! conjugated C in guanidine/guanidinium
MASS    344 NG2P1    14.00700   ! N for protonated imine/Schiff's base (C=N(+)H-R, acyclic amidinium, guanidinium)
MASS   345 NG2P_0    14.007000
MASS   346 HGP2_0     1.008000
MASS   347 HGP2_1     1.008000
MASS   348 HGP2_2     1.008000
MASS   349 NG2P_1    14.007000
AUTO ANGLES DIHE

RESI GUAN  1.000
ATOM C      CG2N1     0.817
ATOM N1     NG2P_0    -0.813
ATOM H1     HGP2      0.437
ATOM H2     HGP2_2     0.437
ATOM N2     NG2P_1    -0.813
ATOM H3     HGP2_0     0.437
ATOM H4     HGP2      0.437
ATOM N3     NG2P1    -0.813
ATOM H5     HGP2      0.437
ATOM H6     HGP2_1     0.437
BOND H6   N3
BOND H5   N3
BOND N1   C
BOND N1   H2
BOND N1   H1
BOND H3   N2
BOND N2   C
BOND N2   H4
BOND C    N3
IMPR C      N1     N2     N3
PATC FIRS NONE LAST NONE
```

```
END

read param card
* MINI FORCE FIELD PARAMETER FILE.
*

BONDS
CG2N1   NG2P1    463.000     1.3650
NG2P1   HGP2     455.000     1.0000


CG2N1    NG2P_0     463.000   1.365
NG2P_0   HGP2       455.000   1.000
NG2P1    HGP2_0     455.000   1.000
NG2P1    HGP2_1     455.000   1.000
NG2P_0   HGP2_2     455.000   1.000
NG2P_1   HGP2_0     455.000   1.000
CG2N1    NG2P_1     463.000   1.365
NG2P_1   HGP2       455.000   1.000


ANGLES
NG2P1   CG2N1   NG2P1      52.000   120.0000    90.000    2.36420
CG2N1   NG2P1   HGP2       49.000   120.0000
HGP2    NG2P1   HGP2       25.000   120.0000




CG2N1    NG2P_0   HGP2       49.000      120.000
CG2N1    NG2P1    HGP2_0     49.000      120.000
NG2P_0   CG2N1    NG2P1      52.000      120.000    90.000    2.36420
HGP2     NG2P_0   HGP2       25.000      120.000
HGP2_0   NG2P1    HGP2       25.000      120.000
CG2N1    NG2P1    HGP2_1     49.000      120.000
HGP2     NG2P1    HGP2_1     25.000      120.000
CG2N1    NG2P_0   HGP2_2     49.000      120.000
```

CG2N1 NG2P_1 HGP2_0 49.000 120.000
CG2N1 NG2P_1 HGP2 49.000 120.000
NG2P_0 CG2N1 NG2P_1 52.000 120.000 90.000 2.36420
HGP2 NG2P_0 HGP2_2 25.000 120.000
NG2P_1 CG2N1 NG2P1 52.000 120.000 90.000 2.36420
HGP2_0 NG2P_1 HGP2 25.000 120.000

DIHEDRALS
NG2P1 CG2N1 NG2P1 HGP2 2.2500 2 180.0
NG2P_0 CG2N1 NG2P1 HGP2_0 0.163582980400664 1 180.0
NG2P_0 CG2N1 NG2P1 HGP2_0 1.870836659750243 2 180.0
NG2P_0 CG2N1 NG2P1 HGP2_0 0.187660256937964 3 0.0
NG2P_0 CG2N1 NG2P1 HGP2_0 0.307892748715624 4 0.0
NG2P_0 CG2N1 NG2P1 HGP2_0 0.051628313328628 6 180.0
NG2P_0 CG2N1 NG2P1 HGP2 2.250000000000000 2 180.0
HGP2 NG2P_0 CG2N1 NG2P1 2.250000000000000 2 180.0
HGP2_0 NG2P1 CG2N1 NG2P1 2.250000000000000 2 180.0
NG2P_0 CG2N1 NG2P1 HGP2_1 0.097285148889586 1 180.0
NG2P_0 CG2N1 NG2P1 HGP2_1 1.993571283530080 2 180.0
NG2P_0 CG2N1 NG2P1 HGP2_1 0.108867143148536 3 0.0
NG2P_0 CG2N1 NG2P1 HGP2_1 0.215264744238450 4 0.0
NG2P_0 CG2N1 NG2P1 HGP2_1 0.040105750865321 6 180.0
NG2P1 CG2N1 NG2P1 HGP2_1 2.250000000000000 2 180.0
NG2P_0 CG2N1 NG2P_1 HGP2_0 0.163582980400664 1 180.0
NG2P_0 CG2N1 NG2P_1 HGP2_0 1.870836659750243 2 180.0
NG2P_0 CG2N1 NG2P_1 HGP2_0 0.187660256937964 3 0.0
NG2P_0 CG2N1 NG2P_1 HGP2_0 0.307892748715624 4 0.0
NG2P_0 CG2N1 NG2P_1 HGP2_0 0.051628313328628 6 180.0
NG2P_0 CG2N1 NG2P_1 HGP2 2.250000000000000 2 180.0
HGP2 NG2P_0 CG2N1 NG2P_1 2.250000000000000 2 180.0
HGP2_2 NG2P_0 CG2N1 NG2P_1 0.024422263034803 1 0.0
HGP2_2 NG2P_0 CG2N1 NG2P_1 1.972971490233108 2 180.0
HGP2_2 NG2P_0 CG2N1 NG2P_1 0.241668160396296 4 0.0
HGP2_2 NG2P_0 CG2N1 NG2P_1 0.043010313701504 6 180.0
HGP2_2 NG2P_0 CG2N1 NG2P1 2.250000000000000 2 180.0
NG2P_1 CG2N1 NG2P1 HGP2_1 2.250000000000000 2 180.0

```
NG2P_1   CG2N1    NG2P1    HGP2      2.250000000000000   2     180.0
HGP2_0   NG2P_1   CG2N1    NG2P1     2.250000000000000   2     180.0
HGP2     NG2P_1   CG2N1    NG2P1     2.250000000000000   2     180.0



IMPROPERS
CG2N1   NG2P1   NG2P1   NG2P1     40.0000        0       0.0
CG2N1    NG2P_0   NG2P1    NG2P1      40.000     0      0.0
CG2N1    NG2P_0   NG2P_1   NG2P1      40.000     0      0.0



NONBONDED  E14FAC  1.000000
HGP2     0.00    -0.046000    0.224500
CG2N1    0.00    -0.110000    2.000000
NG2P1    0.00    -0.200000    1.850000

NG2P_0     0.00   -0.2000    1.8500
HGP2_0     0.00   -0.0460    0.2245
HGP2_1     0.00   -0.0460    0.2245
HGP2_2     0.00   -0.0460    0.2245
NG2P_1     0.00   -0.2000    1.8500

END
```

TMA.str

```
read rtf card

MASS   258 HGA3     1.00800  ! alphatic proton, CH3
MASS   267 HGP2     1.00800  ! polar H, +ve charge
MASS   323 CG334   12.01100  ! aliphatic C for methyl group (-CH3), adjacent to positive N (PROT
NTER) (+)
```

```
MASS   364 NG3P3   14.00700  ! primary NH3+, phosphatidylethanolamine

AUTO ANGLES DIHE


RESI TMA  1.000

ATOM N      NG3P3    -0.357
ATOM C      CG334    -0.008
ATOM H1     HGP2      0.343
ATOM H2     HGP2      0.343
ATOM H3     HGP2      0.343
ATOM H4     HGA3      0.112
ATOM H5     HGA3      0.112
ATOM H6     HGA3      0.112

BOND H1   N    ! 1
BOND H6   C    ! 1
BOND H5   C    ! 1
BOND N    C    ! 1
BOND N    H3   ! 1
BOND N    H2   ! 1
BOND C    H4   ! 1

END



read param card

* MINI FORCE FIELD PARAMETER FILE.

*

BONDS
CG334   NG3P3     200.000     1.4800
CG334   HGA3      322.000     1.1110
```

```
NG3P3   HGP2     403.000     1.0400

ANGLES

NG3P3   CG334   HGA3      45.000   107.5000    35.000    2.10100
HGA3    CG334   HGA3      35.500   108.4000     5.400    1.80200
CG334   NG3P3   HGP2      30.000   109.5000    20.000    2.07400
HGP2    NG3P3   HGP2      44.000   109.5000

DIHEDRALS

HGA3    CG334   NG3P3   HGP2      0.0900        3       0.0

IMPROPERS

NONBONDED  E14FAC  1.000000

HGA3     0.00    -0.024000    1.340000
HGP2     0.00    -0.046000    0.224500
CG334    0.00    -0.077000    2.215000   0.00    -0.010000    1.900000
NG3P3    0.00    -0.200000    1.850000


END
```

MA.str

```
read rtf card
MASS   258 HGA3     1.00800  ! alphatic proton, CH3
MASS   267 HGP2     1.00800  ! polar H, +ve charge
MASS   323 CG334   12.01100  ! aliphatic C for methyl group (-CH3), adjacent to positive N (PROT
NTER) (+)
```

```
MASS   364 NG3P3   14.00700  ! primary NH3+, phosphatidylethanolamine
AUTO ANGLES DIHE

RESI MTHA  1.000
ATOM N      NG3P3    -0.357
ATOM C      CG334    -0.008
ATOM H1     HGP2      0.343
ATOM H2     HGP2      0.343
ATOM H3     HGP2      0.343
ATOM H4     HGA3      0.112
ATOM H5     HGA3      0.112
ATOM H6     HGA3      0.112
BOND H1   N    ! 1
BOND H6   C    ! 1
BOND H5   C    ! 1
BOND N    C    ! 1
BOND N    H3   ! 1
BOND N    H2   ! 1
BOND C    H4   ! 1
PATC FIRS NONE LAST NONE

END

read param card
* MINI FORCE FIELD PARAMETER FILE.
*

BONDS
CG334   NG3P3     200.000     1.4800
CG334   HGA3      322.000     1.1110
NG3P3   HGP2      403.000     1.0400

ANGLES
NG3P3   CG334   HGA3      45.000   107.5000    35.000    2.10100
HGA3    CG334   HGA3      35.500   108.4000     5.400    1.80200
CG334   NG3P3   HGP2      30.000   109.5000    20.000    2.07400
HGP2    NG3P3   HGP2      44.000   109.5000
```

DIHEDRALS

```
HGA3    CG334   NG3P3   HGP2      0.0900        3       0.0
```

IMPROPERS

NONBONDED E14FAC 1.000000

```
HGA3     0.00    -0.024000    1.340000
HGP2     0.00    -0.046000    0.224500
CG334    0.00    -0.077000    2.215000   0.00    -0.010000    1.900000
NG3P3    0.00    -0.200000    1.850000
```

END
